# The predictive significance of umbilical cord bilirubin and bilirubin/albumin ratio for neonatal jaundice in healthy term newborns

**DOI:** 10.55730/1300-0144.5611

**Published:** 2022-12-03

**Authors:** Handan ŞAHAN, Selvi GÜLAŞI, M. Kurthan MERT, Eren K. ÇEKİNMEZ

**Affiliations:** Department of Pediatrics, Faculty of Medicine, Health Sciences University, Adana City Training and Research Hospital, Adana, Turkey

**Keywords:** Cord bilirubin, hyperbilirubinemia, jaundice, phototherapy, newborn

## Abstract

**Background/aim:**

The aim of this study is to determine the value of the questions asked in routine follow-up, the cord blood bilirubin (CBB) and bilirubin/albumin (B/A) ratio in estimating the risk of developing hyperbilirubinemia.

**Materials and methods:**

Term and healthy 217 newborns whose CBB and albumin could be obtained and whose needed to be measured bilirubin level at the 24th and 72nd hours of life were included. Nutrition, sex and nationality, consanguinity between parents, jaundice in the sibling (s), mother’s medications were questioned. CBB and albumin, serum total bilirubin (STB), serum albumin and transcutaneous bilirubin (TcB) at the 24th and 72nd hours of life, were recorded.

**Results:**

CBB and cord B/A ratio, STB and serum B/A ratio, and TcB at the 24th and 72nd hours were found to be higher in the babies who received the phototherapy (p < 0.001 for all). The moderate positive correlation (correlation coefficient 0.383) at the 24th hour and strong positive correlation (correlation coefficient 0.759) at the 72nd hour between STB and TcB measurements was detected. In estimating the need for phototherapy the sensitivity and specificity of CBB were 74.2% and 56.5%, the sensitivity and specifity of cord B/A was 74.2%, and 61.8%. The cut-off value of CBB in estimating the need for phototherapy is 1.8, and the cut-off value of the cord B/A ratio is 0.56. When the cut-off value is 1.8 for the CCB and the cord B/A ratio is 0.56, the positive predictive values are low, but the negative predictive values are significantly high (92.9% and 93.5%, respectively) in determining the need for phototherapy.

**Conclusion:**

CBB and B/A ratio are important in predicting the possibility of indirect hyperbilirubinemia (IHB) development. Babies should be followed closely in terms of IHB development when their CBB value is 1.8 mg/dL and above, and the cord blood B/A ratio is 0.56 and above.

## 1. Introduction

In indirect hyperbilirubinemia (IHB), the aim is to prevent excessive bilirubin levels to eliminate the risk of neurological damage. Each newborn should be evaluated for the risk of IHB before discharge. Guidelines for managing neonatal jaundice use age in hours and other risk factors to determine thresholds for hyperbilirubinemia (HB). But, HB can be noticed only after very high bilirubin concentrations in early discharged infants.

Transcutaneous bilirubin (TcB) measurement is increasingly used in healthy infants, especially in clinics with early discharge, to reduce the need for laboratory bilirubin measurements. But, subcutaneous measurements cannot provide a high-quality evaluation as laboratory tests do. In ideal bilirubin measurement, neonates at risk should be detected with strong accuracy, the test should be inexpensive and noninvasive, and should not require extra equipment. Cord blood bilirubin (CBB) provides most of these properties.

Serum albumin level is important in infants with IHB who require treatment. Low serum albumin may increase the risk of free bilirubin (FB)-induced brain injury by limiting the albumin-bilirubin binding capacity. The bilirubin/albumin (B/A) ratio is related to unbound bilirubin. In order to evaluate the need for exchange transfusion, serum albumin measurement and calculation of the B/A ratio may be used [1].

There have been reports examining the importance of CBB in predicting IHB development, but there are few studies on whether the B/A ratio has value in predicting the development of IHB.

This study aims to evaluate the role of routine follow-up questioning and the bilirubin and B/A ratio from the umbilical cord in demonstrating the risk of IHB development without additional research.

## 2. Materials and methods

This prospective cohort study was held between January 2019 and January 2021 at the University of Health Sciences, Adana City Training and Research Hospital. The ethics committee approval was taken from Adana City Training and Research Hospital (Date: 19.12.2018, Decision no: 364). Written consent was taken from all the families of the infants included in the study.

Since infants born by normal vaginal route were discharged from the hospital in the first 24 h, only babies born by cesarean section could be included in the study.

The criteria for inclusion in the study were determined as healthy and term delivery by cesarean section, having an Apgar score of ≥ 8, having the approval of the family, obtaining cord blood during delivery, and coming to the outpatient clinic on the 3rd day of delivery. Babies with an Apgar score < 8, with congenital malformation, congenital metabolic disease or syndrome, sepsis, asphyxia, intrauterine growth retardation, small for gestational age, and presence of extravascular hemorrhage, which may predispose to hyperbilirubinemia were excluded from the study. The babies born from the mothers with maternal diabetes and those who did not come to the outpatient clinic control at the third day were also excluded. During the study period, the number of babies born by elective cesarean section was 1642, and cord blood was taken from 747 babies. Of these infants, 217 met the inclusion criteria. Eleven pregnant women who were administered oxytocin but could not achieve vaginal delivery and delivered by cesarean section were also included in the study.

Gestational age and birth weight of babies, nutritional quality (breast milk, formula or breast milk and formula), bilirubin and albumin values in cord blood and at the 24th and 72nd hours of life, baby’s sex and nationality, whether there is consanguinity between mother and father, history of jaundice in the previous sibling (s), mother-baby blood groups, direct Coomb’s test positivity, and medications used by the mothers were questioned. Serum total bilirubin (STB) was measured in babies with cord blood taken and in babies with jaundice in the routine examination performed in the first 24 h of life, and these babies were called for outpatient control on the 3rd day.

Patients who have ≥ 80% of the entire amount of daily enteral nutrition with breast milk are grouped as “fed with breast milk,” those who have over 50% of the formula are “fed with formula” those who do not meet the criteria for breast milk and formula are “fed with breast milk + formula”.

Bilirubin was measured by the photometric method (Beckman Coulter AU5800, Inc. USA), and serum albumin concentration was measured by the bromocresol green binding method (Beckman Coulter, Beckman Coulter Inc., USA). Transcutaneous bilirubin meter (MBJ20 Transcutaneous Jaundice Detector, Beijing M&B Electronic Instruments Co., Ltd., China) was used, and measurements were taken from two different points of the baby (forehead and chest), and their average was recorded. Approximately 3–5 mL of cord blood was taken from the placental end into two separate tubes after the cord was clamped. In cord blood, bilirubin, albumin, blood group, and direct Coombs tests were studied.

Based on the Bhutani nomogram, the patients were studied in three groups: bilirubin < 5 (low-risk zone), 5–8 (intermediate risk zone), and ≥ 8 (high-risk zone) at 24 h of life. Cord bilirubin, albumin, and B/A ratio of babies with bilirubin < 12, bilirubin between 12 and 16, and ≥ 16 at 72–96 h were compared.

### 2.1. Statistical analysis

SPSS (IBM Corporation, Armonk, NY, USA) for Windows 20.0 was used for statistical analysis. Data about continuous variables were expressed in mean ± standard deviation if otherwise is not indicated. Intergroup comparisons were made with Student’s t-test (in data with a normal distribution) or with Mann-Whitney U test (in data without normal distribution). Categorical variables were compared with the chi-square test. Pearson’s correlation coefficient was used for continuous variables with normal distribution, and Spearman’s correlation coefficient was used for continuous variables that are not normally distributed p < 0.05 was considered significant.

## 3. Results

The mean gestational age of 217 babies included in the study was 38.0 ± 0.9, and birth weight was 3463.6 ± 365.4 g. One hundred thirteen of the babies (52%) were male. Fifty babies (23%) are refugees, all of them were Syrian. The parents of 90 (41%) babies were second or third-degree relatives, 34 (16%) of the mothers were using drugs (three of them were levothyroxine, the others were ampicillin derivatives), and 52 babies had a history of jaundice in their sibling (s). One hundred thirty babies were predominantly fed by breast milk (60%), 50 infants by formula (23%), and 37 infants (17%) were fed with breast milk and formula. Sixteen (7.4%) of the babies received phototherapy treatment at the 24th hour, 22 (10.1%) at the 72nd hour, and 7 (3.22%) had phototherapy started at the 24th hour and continued at 72 h. The presence of ABO and Rh incompatibility was 25 (12%) and 16 (7%), respectively. Direct Coomb’s positivity was detected in 6% (12) of the group.

Cord bilirubin, B/A ratio, STB and B/A ratio, and TcB at the 24th hour, and STB, B/A ratio, and TcB values at the 72nd hour were found to be significantly higher in the babies who received the phototherapy (p < 0.001 for all) (Table 1).

When patients with low (< 5), moderate (5–8), and high (≥ 8) bilirubin values at the 24th hour were compared, it was determined that patients in the intermediate-risk group had higher cord bilirubin and B/A value than those in the low-risk group (p = 0.001, p = 0.001, respectively). When patients with low (<12), moderate (12–16), and high (≥ 16) bilirubin values at the 72nd hour were compared, it was determined that patients in the intermediate-risk group had higher cord bilirubin and B/A value than those in the low-risk group (p = 0.012, p = 0.012, respectively) (Table 2).

When the correlation between serum total bilirubin and TcB measurements is examined; moderate positive (correlation coefficient 0.383) at the 24th^h^ hour and strong positive (correlation coefficient 0.759) at the 72nd hour was detected. As a result, as STB increases, TcB increases (Figure).

In estimating the need for phototherapy the sensitivity and specificity of cord bilirubin were 74.2% and 56.5%, the sensitivity of cord B/A was 74.2%, and specificity was 61.8%. According to this, the cut-off value of cord bilirubin in estimating the need for phototherapy is 1.8, and the cut-off value of the cord B/A ratio is 0.56. Cord albumin level was ineffective for determining the need for phototherapy (Table 3). When the cut-off value is 1.8 for the cord bilirubin and the cord B/A ratio is 0.56, the positive predictive values are low (22.1% and 24.5%, respectively), but the negative predictive values are significantly high (92.9% and 93.5%, respectively) in determining the need for phototherapy.

## 4. Discussion

Newborn babies are discharged early because of advantages such as preventing nosocomial infections, strengthening the mother-infant bond, and lower cost. Early discharge is more common, especially in regions with high birth rates. Therefore, it is necessary to define markers that predict the possible risk for IHB.

In order to predict the IHB, studies have been carried out evaluating cord bilirubin and first-day bilirubin. In the study of Bhutani et al. [2], bilirubin was examined before discharge, but it was thought to be an invasive method. TcB correlates with STB but sometimes level detected by TcB is lower than STB. Therefore, using TcB measurements in STB nomograms is not recommended [3].

Using CBB is practical, inexpensive, and noninvasive. Jones et al. [4] evaluated 1411 term babies retrospectively and stated that CBB value could predict the development of IHD in infants whose mothers were 0rh+ and 0rh−. In a study by Sun et al. [5], the cut of the value of CBB as 35 μmol/L (approximately 2 mg/dL) was found to predict the development of IHB with a sensitivity of 68.27% with a positive predictive value of 45.68%. In our study, when the cut-off value was 1.8 mg/dL for cord bilirubin, the positive predictive value was low (22.1%). but, the negative predictive value was significantly high (92.9%) in determining the need for phototherapy.

Bilirubin/albumin ratio can help the clinician to evaluate the bilirubin binding capacity of albumin [6]. Free bilirubin (FB) crosses the blood-brain barrier and causes neurotoxicity. Since few approved instruments are used in measurement of FB in routine practice (Arrows UB analyzer UA-1, Arrows Company, Ltd, Osaka, Japan), the B/A ratio may be a surrogate parameter in evaluating free bilirubin levels. Retrospective data considered a high B/A ratio to be a risk factor for bilirubin-induced neurotoxicity. Free bilirubin concentrations depend on STB concentrations, B/A ratio, and bilirubin-albumin binding affinity. Ahlfors et al. [7] mentioned that the B/A ratio is a more appropriate parameter for estimating FB since no test measures FB. STB measurement includes two of the three factors that link with albumin. Sato et al. [8] noted that the B/A ratio was significantly associated with serum-free bilirubin concentration in infants older than 35 weeks of gestation, with serum-free bilirubin of 0.6 mg/dL in concordance with a B/A ratio of 0.5. According to the results, the B/A ratio can predict the FB level when serum-free bilirubin cannot be measured; it is stated that the sensitivity of the B/A ratio in showing serum-free bilirubin is 51%, and the specificity is 99%. The authors of the study emphasized that they achieved a low sensitivity; but, attention should be paid to newborns with FB ≥ 0.6 mg/dL and B/A ratio ≥ 0.5. In their study, Sharma et al. [9] examined the value of CBB, albumin, and B/A ratio in predicting the development of IHB in 388 healthy term newborns born by cesarean section; a cut-off value of 1.9 mg/dL for CBB had a positive predictive value of 71.09%, a sensitivity of 97.4% and a specificity of 40.6%, a cut-off value of 0.719 for cord B/A is 79.6 % of positive predictive value, the sensitivity was 97.4% and the specificity was 62.6%. In our study, when the cord B/A ratio is 0.56, the positive predictive value was low (24.5%), but the negative predictive value is significantly high (93.5%), the sensitivity is 74.2%, and the specificity is 61.8%.

It is known that hereditary characteristics and environmental conditions play a critical role in hyperbilirubinemia, and risk factors are different for each society. In the East Asia, the rate of nonphysiological neonatal jaundice was increased threefold [10]. In a study conducted in our country, G6PD deficiency was described to be 3.8% in newborns with jaundice in the Marmara Region and 8.3% in Çukurova region [11]. Our country is in the G6PD deficiency zone, and its frequency in our country is between 4% and 9%. Castillo et al. [12] developed a simple model using CBB, a noninvasive test, and gestational age and maternal Asian race, two clinical risk factors easily obtained from the medical chart, to predict severe IHB.

In our study, we did not observe an increase in the development of IHB in consanguinity and a sibling (s) with a history of jaundice. We did not find any difference between being a refugee and developing jaundice in IHB development. Thus, we could assume that consanguinity and racial characteristics did not affect cord bilirubin for this study.

It is known that neonatal IHB is more common in breastfed infants than in formula-fed infants. In our study, nutritional quality was not related to the development of IHB at 24 and 72 h. But, late breast milk jaundice occurs between third and fifth day. As our study was limited to the first three days, the effect of late breast milk jaundice on the results could be excluded.

In the study examining the effects of maternal drugs on STB in the newborn baby, phenobarbital, aspirin, phenytoin, chloral hydrate, and meperidine usage was shown to affect bilirubin levels independent from the gestational age, birth weight, sex, breastfeeding, hypoxia, major blood group incompatibility and positive Coombs test [13]. Our study did not detect an increased risk of IHB in the infants of 11 mothers given oxytocin, but this result may be misleading as the number is small.

The early studies on CBB measurement in Rh and ABO incompatibility were recorded in the 1950s. Robinson et al. [14] described that CBB levels above 3 mg/dL could suggest ABO hemolytic disease. Risemberg et al. [15] stated the predictive value of CBB. They suggested that newborns with ABO incompatibility and with cord bilirubin levels greater than 4 mg/dL should be placed in a “high-risk” category. In 1978, Haque et al. [16] reported that CBB was unreliable in predicting hyperbilirubinemia in ABO incompatibility. In a study presented by Whyte et al. [17], CBB was defined as a less reliable indicator but to be more reliable if used with a direct antiglobulin test. In our study, although the mean CBB, cord blood B/A ratio, bilirubin, and B/A ratio values at 24 and 72 h were higher in the group with ABO incompatibility, these differences were not statistically significant.

According to the TcB and/or STB values measured by the postnatal age (hours) before discharge, the risk of developing IHB in the infants is calculated by using the Bhutani nomogram. In a study of Bhutani et al. [18] in 1097 newborns, daily STB values were assessed for five days. IHB was not detected on the 5th day in newborns with an STB value below 5 mg/dL measured between the 20th and 28th hours, and IHB was observed in 33% of the cases whose STB value was at least 8 mg/dL at the same time. In our study, CBB values were found to be statistically significantly lower in patients who were in the intermediate and low risk zone at the 24th hour according to the Bhuthani nomogram, while albumin levels were found to be similar. In our study, the average cord blood albumin level of infants requiring phototherapy in the first 72 h was above 3 g/dL, and there was no infant with albumin below 2.5 g/dL. Conforming to this, it can be thought that the CBB level has a significant predictive value in determining the development of IHB in healthy term babies, and no need to be evaluated together with the albumin level.

Transcutaneous bilirubin measurement can continuously monitor neonatal jaundice, but clinical studies indicate that results are affected by race, skin color, having phototherapy, and instrument error. The reliability of TcB in predicting STB levels decreases when the measurement exceeds 12 mg/dL, in small preterm infants, in term infants after seven postnatal days, and in infants receiving phototherapy [19]. Wainer et al. [20] evaluated the TcB measuring device and reported that TcB measurement in light and medium skin-toned infants correlated well with STB concentrations, but there was a tendency for lower reading in lighter skin tone infants and higher reading in the darker skin tone group. Although TcB results measured with different instruments are less consistent, many studies report that TcB measurement is reliable in terms and infants older than 28 weeks [21]. In our study, a moderately positive correlation was noticed between the STB value measured at the 24th hour and TcB and a strong positive correlation was found between the STB value measured at the 72nd hour and TcB predicting as STB increases, TcB increases, but this relationship may not always be strong.

This study has some limitations. Since most of the pregnant women are not followed up, it is not known whether anti-D immunoglobulin was administered in the previous pregnancy. In addition, since the babies were followed for the first 3 days, we do not know the status of phototherapy after 3 days.

As a result, CBB and cord blood B/A ratio are important in predicting the possibility of IHB development, babies should be followed closely in terms of IHB development when their CBB value is 1.8 mg/dL and above, and the cord blood B/A ratio is 0.56 and above. It has been shown that although its sensitivity is low, its specificity is significantly high, and cord blood albumin level alone does not have a predictive value in healthy infants.

## Figures and Tables

**Figure:**
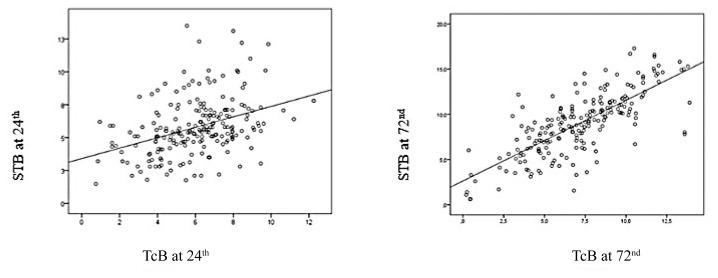
Correlation between STB and TcB value measured at the 24th and 72nd hours.

**Table 1:** Comparison of blood values of infants requiring treatment.

	Phototherapy (+) (n=31)	Phototherapy (−) (n=186)	p
Cord bilirubin (mg/dL)	2.13 ± 0.45	1.76 ± 0.51	**< 0.001**
Cord albumin (g/dL)	3.25 ± 0.35	3.29 ± 0.31	0.539
cord B/A ratio (mg/g)	0.65 ± 0.14	0.54 ± 0.17	**< 0.001**
24th hour STB (mg/dL)	8.44 ± 2.68	5.36 ± 1.64	**< 0.001**
24th hour albumin (g/dL)	3.32 ± 0.25	3.33 ± 0.24	0.745
24th hour B/A ratio (mg/g)	2.54 ± 0.80	1.60 ± 0.48	**< 0.001**
24th hour TcB	5.78 ± 1.99	7.51 ± 1.77	**< 0.001**
72rd hour STB (mg/dL)	13.72 ± 2.30	8.22 ± 2.85	**< 0.001**
72rd hour albumin (g/dL)	3.41 ± 0.24	3.45 ± 0.28	0.411
72rd hour B/A ratio (mg/g)	4.03 ± 0.70	2.39 ± 0.84	**< 0.001**
72rd hour TcB	10.21 ± 2.25	6.64 ± 2.64	**< 0.001**

Values are calculated as mean ± standard deviation.

**Table 2:** Comparison of blood values of infants at 24th and 72nd hours.

	24th hour STB < 5 (n=68)	24th hour STB 5–8 (n=122)	24th hour STB ≥ 8 (n=27)	p
Cord bilirubin (mg/L)	1.57 ± 0.41	1.9 ± 0.53	2.01 ± 0.51	**< 0.001**^a^,^b^
Cord albumin (g/dL)	3.29 ± 0.23	3.28 ± 0.35	3.3 ± 0.36	0.957
Cord B / A ratio (mg/g)	0.48 ± 0.13	0.58 ± 0.18	0.62 ± 0.14	**< 0.001**^a^,^b^
	**72rd hour STB < 12 (n=180)**	**72rd hour STB 12–16 (n=33)**	**72rd hour STB ≥ 16 (n=4)**	**p**
Cord bilirubin (mg/dL)	1.76 ± 0.52	2.04 ± 0.46	2.01 ± 0.43	**0.012** c
Cord albumin (g/dL)	3.29 ± 0.31	3.25 ± 0.35	3.24 ± 0.11	0.716
Cord B / A ratio (mg/g)	0.54 ± 0.17	0.63 ± 0.15	0.62 ± 0.15	**0.012** c

Values are calculated as mean ± standard deviation.

a for the 24th hour STB < 5 and the 24th hour STB 5–8 p < 0.05,

b for the 24th hour STB < 5 and the 24th hour STB ≥ 8, p < 0.05,

c for the 72nd hour STB < 12 and the 72nd hour STB12–16, p < 0.05.

**Table 3:** The importance of cord bilirubin and albumin in determining the need for phototherapy.

	Cut-off (mg/dL)	Sensitivity (%)	Specificity (%)	PPV (%)	NPV (%)
Cord bilirubin (mg/dL)	1.8	74.2	56.5	22.1	92.9
Cord albumin (g/dL)	NA – not good at distinguishing				
Cord B/A ratio (mg/g)	0.56	74.2	61.8	24.5	93.5

PPV: positive predictive value, NPV: negative predictive value.
